# Correction: SCF^β-TRCP^ suppresses angiogenesis and thyroid cancer cell migration by promoting ubiquitination and destruction of VEGF receptor 2

**DOI:** 10.1084/jem.2011244608082025c

**Published:** 2025-08-18

**Authors:** Shavali Shaik, Carmelo Nucera, Hiroyuki Inuzuka, Daming Gao, Maija Garnaas, Gregory Frechette, Lauren Harris, Lixin Wan, Hidefumi Fukushima, Amjad Husain, Vania Nose, Guido Fadda, Peter M. Sadow, Wolfram Goessling, Trista North, Jack Lawler, Wenyi Wei

Vol. 209, No. 7 | https://doi.org/10.1084/jem.20112446 | June 18, 2012

The authors regret that, during figure preparation, the tubulin blot from Fig. 7 A was inadvertently used in Fig. 4 A. The original and revised Fig. 4 are shown here. This error does not affect the results and conclusions of the study, and the figure legend remains unchanged. The error appears in print and in PDFs downloaded before August 8, 2025.

**Figure:**
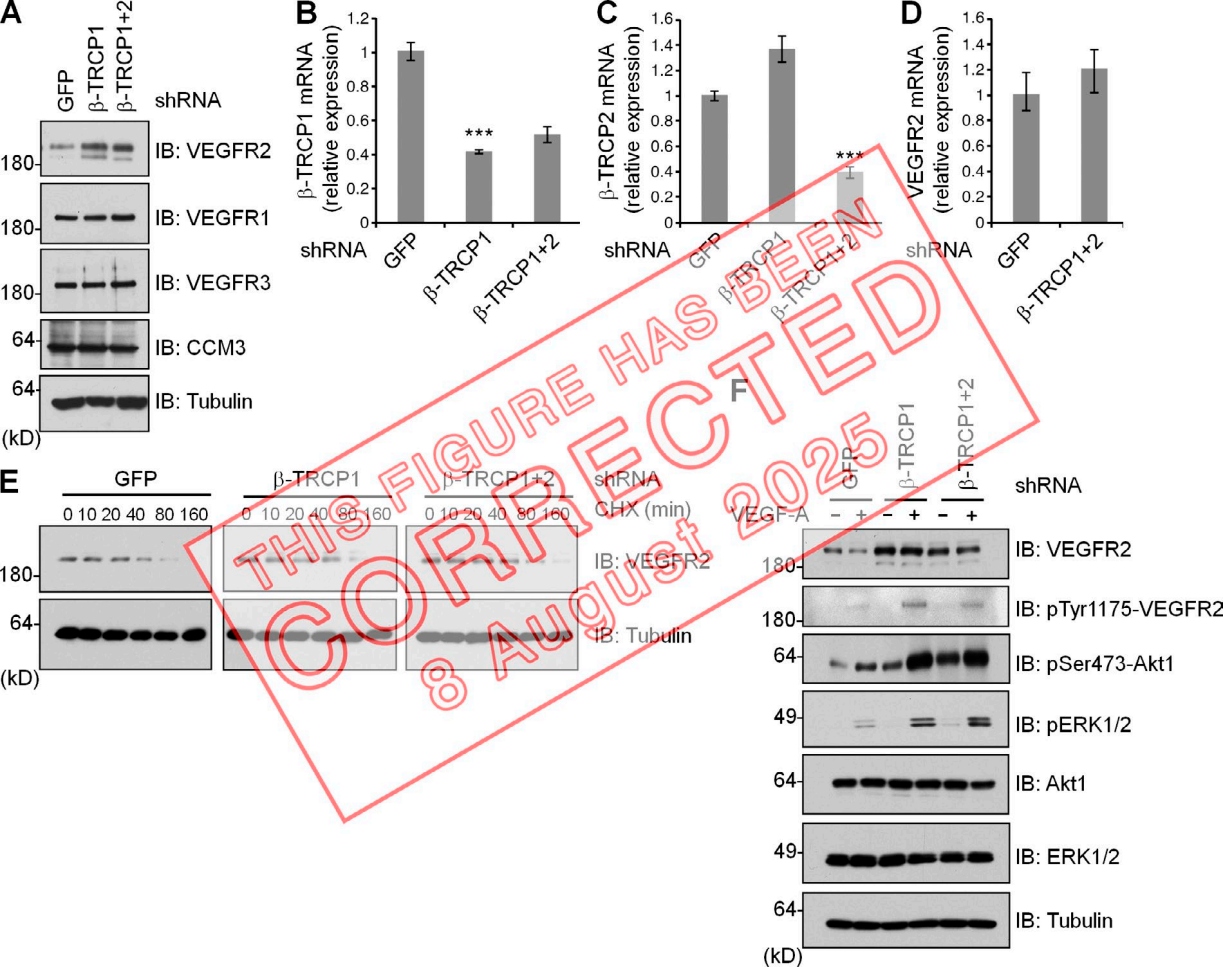


**Figure 4:**
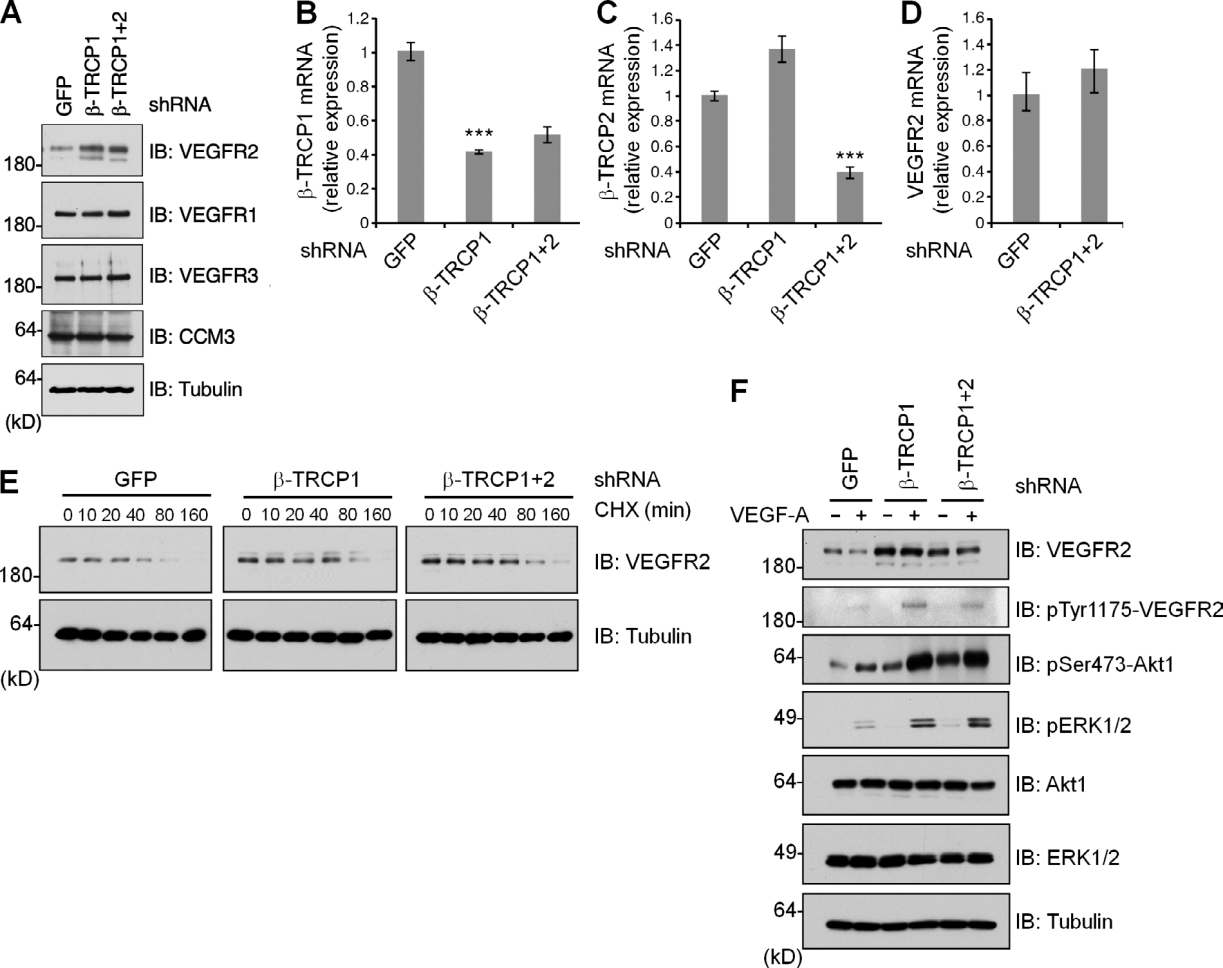
**β-TRCP regulates VEGFR2 protein levels in HMVECs. (A)** Immunoblot analysis of HMVECs stably expressing lentiviral vectors encoding shRNA specific for GFP, β-TRCP1, and β-TRCP1+2. Data shown is representative of two independent experiments. **(B and C)** Real-time RT-PCR analysis of β-TRCP1 (B) and β-TRCP2 (C) mRNA expression in HMVECs infected with the indicated shRNA lentiviral vectors. Error bars indicate mean ± SD from three independent experiments. ***, P < 0.001. **(D)** Real-time RT-PCR analysis of VEGFR2 mRNA expression in HMVECs infected with the indicated shRNA lentiviral vectors. Error bars indicate mean ± SD from three independent experiments. **(E)** HMVECs were infected with the indicated shRNA lentiviral vectors. After selection with 1 µg/ml puromycin for 72 h, cells were split into 60-mm dishes and, after another 20 h, treated with 20 µg/ml CHX. At the indicated time points, WCLs were prepared, and immunoblots were probed with the indicated antibodies. Data shown is representative of three independent experiments. **(F)** Immunoblot analysis of the HMVECs shown in A, stimulated where indicated with 100 ng/ml VEGF-A for 15 min. Data shown is representative of two independent experiments.

